# Microbial Electrochemical Fluidized Bed Reactor: A Promising Solution for Removing Pollutants From Pharmaceutical Industrial Wastewater

**DOI:** 10.3389/fmicb.2021.737112

**Published:** 2021-11-26

**Authors:** Yeray Asensio, María Llorente, Alejandro Sánchez-Gómez, Carlos Manchon, Karina Boltes, Abraham Esteve-Núñez

**Affiliations:** ^1^Departamento de Química Analítica, Química Física e Ingeniería Química, Universidad de Alcalá, Alcalá de Henares, Spain; ^2^IMDEA Water Institute, Alcalá de Henares, Spain

**Keywords:** fluidized bed, emerging contaminants, pharmaceutical compounds removal, wastewater treatment, electroactive bacteria, microbial electrochemical technologies

## Abstract

The capacity of electroactive bacteria to exchange electrons with electroconductive materials has been explored during the last two decades as part of a new field called electromicrobiology. Such microbial metabolism has been validated to enhance the bioremediation of wastewater pollutants. In contrast with standard materials like rods, plates, or felts made of graphite, we have explored the use of an alternative strategy using a fluid-like electrode as part of a microbial electrochemical fluidized bed reactor (ME-FBR). After verifying the low adsorption capacity of the pharmaceutical pollutants on the fluid-bed electrode [7.92 ± 0.05% carbamazepine (CBZ) and 9.42 ± 0.09% sulfamethoxazole (SMX)], our system showed a remarkable capacity to outperform classical solutions for removing pollutants (more than 80%) from the pharmaceutical industry like CBZ and SMX. Moreover, the ME-FBR performance revealed the impact of selecting an anode potential by efficiently removing both pollutants at + 200 mV. The high TOC removal efficiency also demonstrated that electrostimulation of electroactive bacteria in ME-FBR could overcome the expected microbial inhibition due to the presence of CBZ and SMX. Cyclic voltammograms revealed the successful electron transfer between microbial biofilm and the fluid-like electrode bed throughout the polarization tests. Finally, *Vibrio fischeri*-based ecotoxicity showed a 70% reduction after treating wastewater with a fluid-like anode (+ 400 mV), revealing the promising performance of this bioelectrochemical approach.

## Introduction

The synthetic chemical industry produces effluents with a high level of salinity and a high chemical oxygen demand (COD), which eventually generates a dispersed pollution problem in the form of micropollutants, also called emerging contaminants (EC) (Queiroz et al., 2019). The pharmaceutical industry produces the most recalcitrant wastewater (Abdel-Shafy and Mansour, 2013; Gadipelly et al., 2014; Nassiri Koopaei and Abdollahi, 2017; Yakubu, 2017). Pharmaceutical wastewater is known to contain highly active compounds, which can lead to a significant concern due to their potential adverse human health and ecological effects (Virkutyte et al., 2010). Some of the most frequently detected pharmaceutical active compounds in wastewater are carbamazepine (CBZ) and sulfamethoxazole (SMX), which are recalcitrant compounds whose consumption has significantly increased in the last decades (García-Espinoza and Nacheva, 2019). In the last years, significant efforts have been made to decrease the concentration of such EC in real wastewaters, attending to the current poor removal rates achieved in conventional biological treatments for industrial effluents as standard anaerobic digestion (Zhang et al., 2008; García-Gómez et al., 2016; Chtourou et al., 2018). Furthermore, it has been suggested that biological treatment is not recommended for treating wastewater with a high concentration of CBZ and SMX since such pharmaceutical compounds can inhibit the biological activity of microorganisms present in conventional activated sludge (CAS) treatments (Li et al., 2013).

Membrane bioreactors (MBR) have been the most evaluated technology for CBZ and SMX removal in synthetic hospital wastewaters, achieving higher detoxification rates, around 37% of CBZ and SMX removal, in comparison with CAS treatments during long-term operation (Hai et al., 2011; García-Gómez et al., 2016; Cecconet et al., 2017; Cheng et al., 2018; Gurung et al., 2018). Furthermore, anaerobic MBR (AnMBR) have also been evaluated, increasing the CBZ and SMX removal up to 80% (Cheng et al., 2018). Nevertheless, operational problems such as membrane biofouling, high costs associated with membranes, and the high energy requirements for both technologies had limited the scale-up of these technologies for treating wastewaters polluted with pharmaceutical compounds (Hai et al., 2011; García-Gómez et al., 2016; Cheng et al., 2018).

Considering the relatively high removal of these pharmaceutical compounds under advanced anaerobic treatments as AnMBR, it seems reasonable to explore technological solutions where microbial communities play a crucial role. In this context, microbial electrochemical technologies (MET) have been recently classified as one the most promising for achieving sustainable bioremediation in a different environmental niche (Wang et al., 2020). MET are based on the redox properties of electroactive bacteria to transfer those electrons released from pollutant metabolism into an electroactive material. This redox coupling eventually results in higher biodegradation rates than electrode-free anaerobic communities from soil, sediments, and freshwater (Liu et al., 2005; Zhang and Angelidaki, 2014; Bajracharya et al., 2016; Domínguez-Garay et al., 2016). Furthermore, MET have been historically evaluated regarding wastewater treatment capacity through three different approaches: microbial fuel cells (MFC) (Logan, 2008; Domínguez-Garay et al., 2016; Cecconet et al., 2018; Gajda et al., 2018; Gao et al., 2020; Lin et al., 2020), microbial electrolysis cells (MEC) (Srikanth et al., 2008; Zhang and Angelidaki, 2014; Scott and Yu, 2015; Bajracharya et al., 2016; Leon-Fernandez et al., 2019), and microbial electrochemical snorkel (MES) (Yang et al., 2013, 2015; Viggi et al., 2017; Hoareau et al., 2019; Pun et al., 2019; Ramírez-Vargas et al., 2019). Among them, dual-chamber MEC has been the most evaluated system for removing pharmaceutical pollutants like CBZ and SMX due to successful performance at lab scale (Harnisch et al., 2013; Rodrigo Quejigo et al., 2019; Tahir et al., 2019). Nevertheless, some bottlenecks, such as the ionic exchange membrane (IEM) cost or a large limitation in mass transfer, have delayed the further scaling of such a system. In contrast to conventional electroconductive materials (graphite bars, plates or granules, and carbon felt), a new configuration based on the use of fluid-like electrodes recently appeared in the MET field (Tejedor-Sanz et al., 2017a). The newborn reactor, microbial electrochemical fluidized bed reactor (ME-FBR), opened the electromicrobiology field to bacteria that do not necessarily form an electroactive biofilm but perform extracellular electron transfer as planktonic cells (Tejedor-Sanz et al., 2017a). The result is a hybrid concept hosting both the optimal mass transfer of fluidized bed reactors and the high biodegradation rate of bioelectrochemical systems. This new concept has already been successfully operated for treating brewery wastewater (Tejedor-Sanz et al., 2016, 2017a; Asensio et al., 2021). In addition, the absence of ionic membranes in ME-FBR decreases further capital costs present in more conventional systems (Tiquia-Arashiro and Pant, 2020).

In contrast with already existing ME-FBR applications (Tejedor-Sanz et al., 2016, 2017a,b; Sara et al., 2020), no previous studies have reported pharmaceutical compound removal using fluid-like electrodes. In this work, we have investigated for the first time the capability of a lab-scale ME-FBR for removing two pharmaceutical compounds, CBZ and SMX, operated in a semi-continuous mode under different anodic potentials. The treated water’s ecotoxicity was also monitored to assure the final quality of the effluents.

## Materials and Methods

### Design and Construction of Bioelectrochemical Reactors: Microbial Electrochemical Fluidized Bed Reactor

The ME-FBR was designed and constructed as previously reported (Tejedor-Sanz et al., 2017a,b, 2018). The tubular ME-FBR had a total volume of 1.2 L, including the recirculation pipe and the electroconductive bed volume. The working electrode (anode) consisted of a bed composed of electroconductive vitreous carbon particles (20% *V*_electroconductive material_/*V*_net reactor volume_) with a diameter range of 0.6–1 mm (Chemviron Carbon ^®^, Belgium). A graphite plate (20 × 80 mm) was vertically immersed into the electroconductive bed material, acting as the current collector.

A Ti/Pt mesh (30 × 40 mm) was used as cathode material (Inagasa, Spain) at the top of the ME-FBR reactor.

The ME-FBR was operated as a three-electrode electrochemical cell. The fluidized bed potential was fixed to different values. A potentiostat (Nanoelectra NEV3, Spain) was used for poisoning the working electrode from −200 to + 600 mV [all anode potentials were reported vs. Ag/AgCl electrode (HANNA)]. Additionally, an electrochemical control test was performed under abiotic conditions. The data logger installed in the NEV3 potentiostat registered the output current during wastewater treatment. The current density values for the ME-FBR are given as current per net reactor volume (NRV). Cyclic voltammograms were performed at a scan rate of 0.005 V s^–1^.

Finally, two control tests (see Supplementary Figures 1, 3B) were performed in reactors with the same architecture and volume as the ME-FBR described above. The first one was an electrochemical control tested under abiotic conditions, while the second control was an electrode-free anaerobic bioreactor (UASB).

### Microbial Growth and Media Composition

Granular anaerobic sludge from a wastewater treatment plant (Guadalajara, Spain) was used as the initial inoculum. This anaerobic sludge was diluted 1:3 with synthetic wastewater. The cell suspension was incubated in the bioelectrochemical reactors for 4 days, without aeration, to encourage the growth of the anaerobic culture. During such inoculation action, no pollutants were supplied to the reactors. After this period, pollutant-spiked wastewater was fed into the reactors.

The synthetic wastewater contained 2.5 g L^–1^ NaHCO_3_, 0.75 g L^–1^ NH_4_Cl, 0.60 g L^–1^ NaH_2_PO_4_, 0.1 g L^–1^, KCl 4.1 g L^–1^ NaC_2_H_3_O_2_, and 0.1 ml L^–1^ mineral stock solution and a vitamin stock solution as previously described in previous work (Tejedor-Sanz et al., 2016). The synthetic wastewater was enriched with two pharmaceutical compounds: CBZ (Sigma Aldrich, CAS 298-46-4) and SMX (Sigma Aldrich, CAS 723-46-6). The standard synthetic wastewater contained 10 and 3 mg L^–1^ of CBZ and SMX, respectively. Nevertheless, to evaluate the biodegradation capacity from the initial inoculum, different solutions of CBZ and SMX were used.

### Microbial Electrochemical Fluidized Bed Reactor Operation

The ME-FBR was fed with synthetic wastewater once a day, so its operation mode can be considered semi-continuous with 3.2 days of hydraulic retention time (HRT). The other parameters, temperature (37°C) and N_2_:CO_2_ (80:20) headspace gas, were kept constant. The electrolyte recirculation velocity used during the experiments was 0.68 cm s^–1^. Effluent samples were taken daily.

### Chemical Analysis

Samples were filtered with 0.45-μm nylon membranes prior to storage at −20°C. TOC was analyzed in both feed stream and effluents after 1:10 dilution using a TOC-VCSH Shimadzu analyzer. Acetate concentration was measured by an HP series 1,100 high-pressure liquid chromatograph coupled with a UV detector (210 nm), equipped with a Supelco C-610H column and using 0.1% H_3_PO_4_ as the mobile phase with a flow rate of 0.5 ml min^–1^. In addition, pH and conductivity were measured daily by a HACH HQ40D ^®^ multiparametric probe to control the technical performance of the anaerobic systems. CBZ and SMX concentrations were measured using an HP series 1,100 high-pressure liquid chromatograph equipped with an automation injection, a diode array detector (280 nm), and a Kromasil column (150 × 4.6 × 1.5 μm).

### Ecotoxicity Bioassays

Measurement of the luminescent decay of the bacteria *Vibrio fischeri* NRRL-B 11177 was used to test synthetic wastewater and treated effluent toxicity. Bioassays were carried out using lyophilized bacteria after their reactivation following instruction from BioFix ^®^ Lumi (Macherey-Nagel, Germany). Tests were conducted at 15°C with minimal modification of the standard ISO 11348-3:2007 to measure light emission in 96-well microplates, using Fluoroskan Ascent FL (Thermo Fisher Scientific).

The impact of pharmaceuticals on the bioluminescence emission of bacteria was measured by triplicate as the inhibition percentage regarding the light emission of the control test. Toxicity values were calculated after 28 min of exposure time.

### Adsorption Assay

The adsorption capacity from a fluid-like electrode to retain CBZ and SMX was assessed by continuously feeding under gravity 50 ml of synthetic wastewater through 10 g of vitreous carbon until the final concentration of the pharmaceutical compounds was stable for three consecutive cycles. Finally, 10 additional feeding steps were performed. Additionally, it is also important to remark the operation of an electrochemical abiotic control test (described in section “Design and Construction of Bioelectrochemical Reactors: Microbial Electrochemical Fluidized Bed Reactor”), which also provided adsorption data under electrode polarization.

## Results and Discussion

Pharmaceutical compounds like CBZ and SMX are recalcitrant pollutants from the pharmaceutical industry; however, our research has revealed that microbial catabolism can be electrochemically stimulated by fluid-like electrodes using an ME-FBR.

### Microbial Anaerobic Biodegradation of the Pharmaceutical Pollutants Carbamazepine and Sulfamethoxazole

**FIGURE 1:**
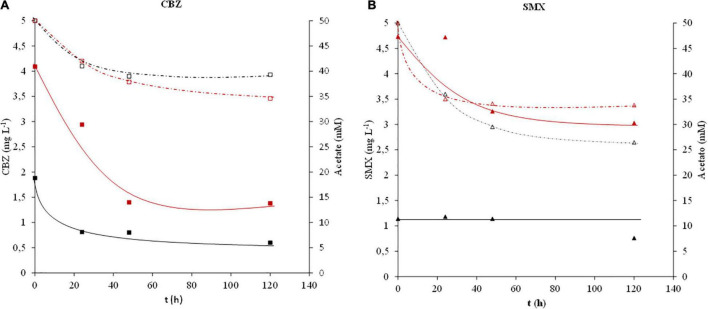
CBZ and SMX removal capacity of the anaerobic consortium. **(A)** CBZ removal capacity: 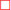 acetate concentration in synthetic wastewater polluted with ∼4 mg L^–1^ CBZ, □ acetate concentration in synthetic wastewater polluted with ∼2 mg L^–1^ CBZ, 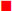 CBZ concentration in 4 mg L^–1^ CBZ-polluted synthetic wastewater, ■ CBZ concentration in 2 mg L^–1^ CBZ-polluted synthetic wastewater. **(B)** SMX removal capacity: 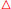 acetate concentration in synthetic wastewater polluted with ∼4.7 mg L^–1^ SMX, △ acetate concentration in synthetic wastewater polluted with ∼1 mg L^–1^ SMX, 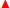 SMX concentration in ∼4.7 mg L^–1^ SMX-polluted synthetic wastewater, ▲ SMX concentration in ∼1 mg L^–1^ SMX-polluted synthetic wastewater.

The anaerobic removal of CBZ and SMX by our microbial consortium was evaluated during 120 h in batch mode. During this assay, electrochemistry was not involved in order to evaluate the capacity of the anaerobic inoculum to remove CBZ and SMX (Figure 1). Our results revealed that CBZ was partially removed by the anaerobic consortium regardless of the concentration tested (4.09 and 1.88 mg L^–1^ of CBZ). On the contrary, anaerobic bacteria were inefficient in biodegrading SMX at low concentrations (1.14 mg L^–1^ of SMX), showing a minor SMX removal capacity at high concentrations (4.73 mg L^–1^ of SMX).

In order to assess the biodegradation capacity of the microbial consortium, the synthetic wastewater was spiked with 50 mM acetate as the sole electron donor in addition to either CBZ or SMX.

Two different stages could be identified during the acetate and pollutant removal. During the first 48 h, anaerobic bacteria simultaneously consumed both the acetate and the pharmaceutical compounds. The removal rates for CBZ were higher (∼1.3 mg L^–1^ day^–1^ of CBZ) when the pollutant was given at the highest doses (∼4 mg L^–1^). The acetate removal during both experiments was ∼20% regardless of the doses of CBZ. After the first stage, no further removal of CBZ or acetate was observed, suggesting a stationary phase.

On the contrary, in the case of SMX-polluted wastewater, a different pattern was observed. A lower SMX removal was achieved compared to CBZ oxidation. However, cells were metabolically active since a higher acetate removal was observed. Thus, wastewater spiked with 4.7 mg L^–1^ of SMX showed a removal of 0.74 mg L^–1^ day^–1^ of SMX in contrast to a null removal after supplying wastewater with ∼1.1 mg L^–1^ SMX.

Low removal of both CBZ and SMX at ∼1 mg L^–1^ pollutant concentration could be related to mass transfer limitations. Nevertheless, CBZ and SMX may not have been entirely mineralized due to the low capacity of conventional anaerobic treatments, as anaerobic digesters, to remove these complex organic molecules (Ali, 2019; Angeles et al., 2019; Mestre and Carvalho, 2019; Pun et al., 2019). CBZ and SMX were expected to be partially oxidized to form intermediates that may not further be converted into methane and carbon dioxide using this conventional anaerobic condition. Usually, intermediates from pharmaceuticals can be more toxic than the initial organic micropollutants, so effective treatments should be performed to reach complete mineralization of pharmaceutical chemicals (Rodrigo et al., 2010; Song et al., 2020). According to previous results, higher metabolic oxidation rates should be achieved in order to remove CBZ and SMX completely.

### Microbial Electrochemical Fluidized Bed Reactor Is an Efficient Technology for Removing Carbamazepine and Sulfamethoxazole From Wastewater

**FIGURE 2:**
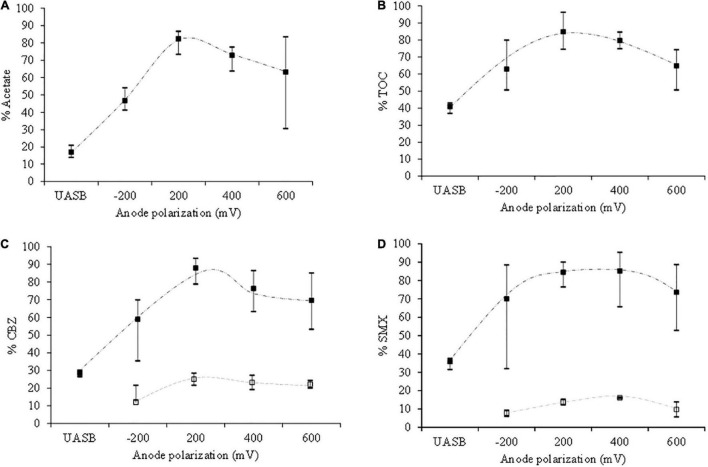
Acetate, TOC, CBZ, and SMX removal in the UASB reactor (no anode potentials) and ME-FBR in a vast range of applied anode potentials (−200, 200, 400, and 600 mV). **(A)** Acetate removal. **(B)** TOC removal. **(C)** CBZ removal. **(D)** SMX removal. Symbols: ■% removal, □% CBZ and SMX removal in electrochemical control test.

Once we demonstrated the natural capacity of our consortium to remove CBZ and SMX, then we proceed to grow such microbial population using an ME-FBR. In order to discard the role of our electroconductive material for retaining CBZ and SMX, we performed a number of assays (Supplementary Figure 1) that showed a minor impact (9.42 ± 0.09 and 7.92 ± 0.05%, respectively). The retention tests were performed in triplicate. In parallel, we designed two key control reactors to (a) evaluate the impact of our material to remove CBZ and SMX under electrochemical operation in the absence of microorganisms and (b) evaluate the biodegradation capacity of our microbial consortium (UASB) in the absence of fluidized electrode. Indeed, CBZ and SMX pollutants were removed just in the range of 14–16% after the abiotic electrochemical control (see Supplementary Figure 3B). Regarding the electrode-free control, our UASB was able to remove just 28% for CBZ and 36% for SMX (Figure 2). In contrast, the full operation of our microbial electrochemical reactor ME-FBR under the semi-continuous mode for ∼30 days outperformed the UASB wastewater treatment capacity in terms of acetate, TOC, CBZ, and SMX removal.

Precisely, the ME-FBR validation was performed under different anode potentials to monitor the removal of acetate, CBZ, and SMX. An optimal removal of all pollutants including acetate, CBZ (−*r* CBZ = 2.75 mg L^–1^ day^–1^ of CBZ; 88% CBZ removal), and SMX (−*r* SMX = 0.79 mg L^–1^ day^–1^ of SMX; 85% SMX removal) was observed when the anode was acting as the electron acceptor at + 200 mV (vs. Ag/AgCl).

Furthermore, the removal was kept in the range of 70–80% at anode potentials as high as + 400 mV. At higher anode polarization, + 600 mV (vs. Ag/AgCl), the TOC removal decreased to 63%, while CBZ and SMX showed a slight decrease compared to the operation at lower anode potentials. The reduced electrobioremediation efficiency at potentials as high as + 600 mV has been previously reported and is most likely due to an alteration in the permeability of the bacterial membrane (Rodrigo Quejigo et al., 2019), resulting in lower microbial viability.

Finally, CBZ and SMX removal was evaluated under an anode potential with a negative value (−200 mV). Under potentiostatic control at negative potential value, organic matter and pharmaceutical compound removal was not favored according to the low TOC removal (63%), including just 50% removal of an easily biodegradable compound like acetate. Based on our results, the optimization of the anode potential is key in order to maximize pollutant removal.

**FIGURE 3:**
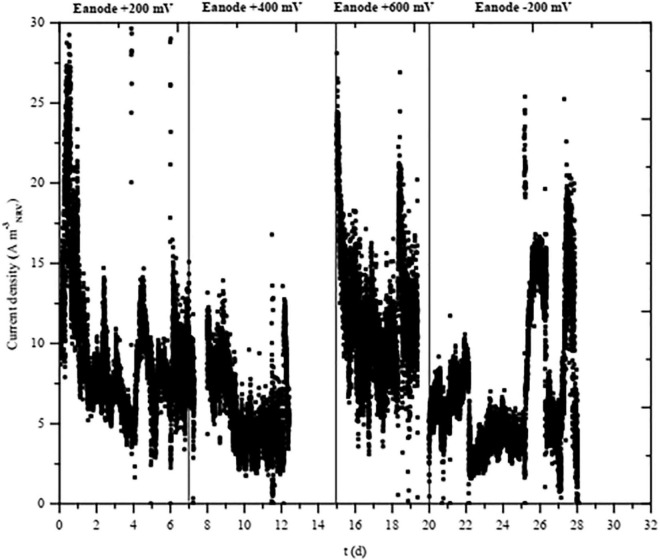
Current density during the long-term operation of the ME-FBR at periods where anode was polarized at the labeled potentials (−200, 200, 400, and 600 mV). Symbols: ■ current density (A m^–3^_NRV_).

During the whole ME-FBR operation, the current density was continuously monitored. It can be observed that at + 400 mV (vs. Ag/AgCl), some current data are missing. During those days, the potentiostat described in section “Materials and Methods” (NEV3) was replaced by a conventional power supply due to operational problems in order to continue polarizing the fluid-like anode. The conventional power supply operated in that period did not include a data logger. A cell-free abiotic assay revealed negligible current density in contrast to values as high as 20 A m^–3^ obtained when electroactive bacteria coupled the oxidation of pollutants and electron transfer to the electroconductive particles. Higher current densities were obtained under the most positive anode, while it was minimized at values as low as −200 mV (Figure 3).

The electrochemical operation of our ME-FBR requires low energy consumption, approximately 2.3 ± 0.4 Wh according to the chronoamperometric figures observed in Figure 3. Additionally, our systems share all the OPEX advantage of any anaerobic treatment performed with our reduced sludge production and with no need of artificial aeration.

An irregular chronoamperometric profile was obtained during the semi-continuous operation of ME-FBR. Current density typically achieved maximum values when TOC was available for electroactive bacteria, while it reached minimum values when TOC was biologically consumed.

**FIGURE 4:**
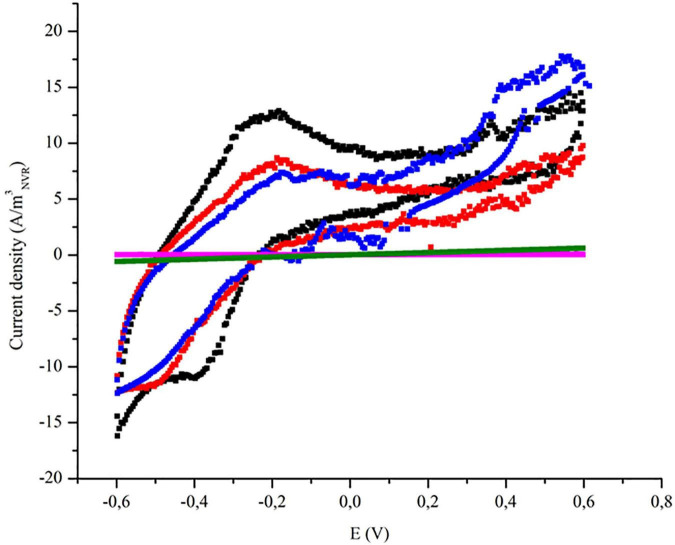
Cyclic voltammograms during different stages where ME-FBR was operated under different anode potentials. Symbols: 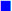 applied anode potential of + 600 mV, ■ applied anode potential of + 400 mV, 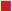 applied anode potential of + 200 mV, 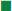 applied anode potential of −200 mV, 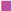 blank—abiotic conditions.

The polarization potential of the fluid-like electrode drove the metabolic activity of the biofilm. In order to study the electrochemical interaction between the microbial community and the electrode, cyclic voltammograms were performed after operating the system under different anode potentials for at least 1 week (Figure 4).

The cyclic voltammograms were performed under catalytic conditions (turnover), and they showed oxidation–reduction peaks that were not present in the cell-free abiotic assays.

At negative potential (−200 mV), the voltammogram showed no peaks, while the electric capacity was significantly less compared to the voltammograms obtained at positive potentials. The absence of peaks pointed out the weak interaction between electroactive bacteria and the fluid-like electrode, according to current density (Figure 3). The low electrical capacity for this electrode was almost identical to the cell-free abiotic assay. Accordingly, the low values for both TOC and antibiotic removal were consistent with the poor electron transfer among bacteria and the fluid-like electrode at −200 mV.

However, this interaction between electroactive bacteria and fluid-like electrode was considerably increased at positive anode potentials (+ 200, +400, and + 600 mV). The higher interaction could be correlated with the width of the obtained voltammograms and the presence of redox peaks during the vast range of tested potentials. Those redox peaks were observed at −500 mV (reduction peak) and −200 mV (oxidation peak), achieving a maximum current density of 13 A m^–3^_NRV_ when the anode was poised at + 400 mV. It is important to note that voltammograms were similar when fluid-like anode was polarized either at + 200 or + 400 mV. In contrast, the voltammogram at + 600 mV (vs. Ag/AgCl) showed redox peaks that suggested less electroactivity in the biofilm. Instead, two new peaks were observed, a reduction peak at −200 mV and an oxidation peak at −400 mV, suggesting a shift in the ETT mechanism previously reported by *Geobacter sulfurreducens* when anode polarization was observed upgraded from + 200 to + 600 mV (Busalmen et al., 2008).

Cyclic voltammogram analysis revealed that interaction between electroactive bacteria and the working electrode was optimized by applying an anode potential of + 400 mV. Nevertheless, CBZ removal was slightly higher at + 200 mV (Figure 2), which suggests that non-electroactive communities from ME-FBR may also have a role in biodegrading certain pharmaceutical pollutants.

### Microbial Electrochemical Fluidized Bed Reactor Is a Step Forward Concerning Conventional Electrochemical Systems for Removing Carbamazepine and Sulfamethoxazole From Wastewater

After optimization of the applied anode potential, the achieved ME-FBR bioremediation was compared with the UASB reactor’s wastewater treatment capacity.

As previously shown, TOC removal was remarkably higher in the ME-FBR case than in the UASB treatment where the anaerobic oxidation capacity of the anaerobic consortium was tested for pharmaceutical compound removal, with a %TOC removal of ∼85%, indicating higher removal of the pharmaceutical compounds compared with the conventional anaerobic treatment (43.18%). Higher TOC removal was obtained with ME-FBR due to (i) the cooperation between an active electrode biofilm and planktonic bacteria not limited to mass transfer issues and (ii) the continuous polarization of the fluidized bed that boosts the pharmaceutical compound removal, compared to conventional anaerobic digestion technologies.

### Toxicity Analysis

**FIGURE 5:**
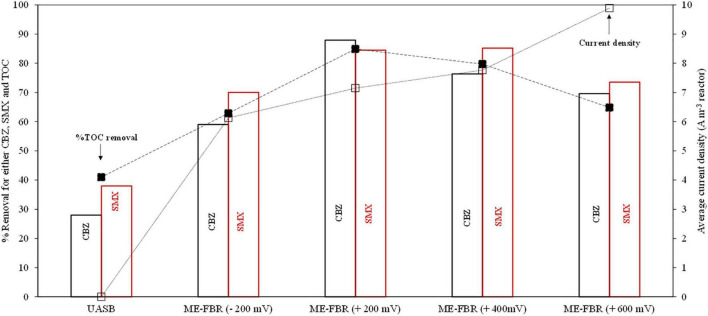
Comparison of the micropollutant and TOC removal of the ME-FBR and the UASB reactor. Symbols: ■% TOC removal, □ average current density (A m^–3^ ME-FBR).

As shown in the preceding sections, CBZ and SMX oxidation led to the formation of chemical intermediate species. This fact is easily observed in the partial removal of TOC (Figures 2, 5). In order to evaluate the potential ecotoxicity of such intermediates, we measured the antibacterial activity of our effluents toward *V. fischeri*.

**FIGURE 6:**
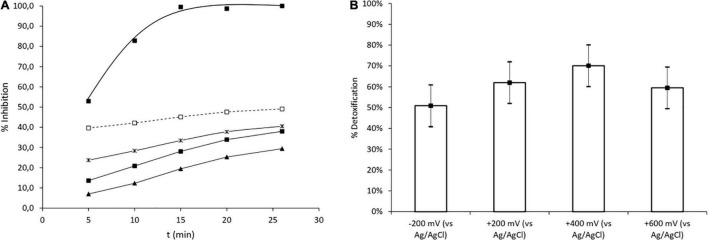
Ecotoxicity test using *V. fischeri*. **(A)** Profile of bioluminescence inhibition during exposure time. **(B)** Detoxification of synthetic wastewater under different anode potentials. Symbols: ■% inhibition of non-treated synthetic wastewater, □ treated wastewater at −200 mV (vs. Ag/AgCl), ▲ treated wastewater at + 400 mV (vs. Ag/AgCl), × treated wastewater at + 600 mV (vs. Ag/AgCl), ■ treated wastewater at + 200 mV (vs. Ag/AgCl).

The luminescence inhibition of the polluted synthetic wastewater reached 100% after 15 min of exposure time, clearly indicating its high ecotoxicity (Figure 6A). That luminescence inhibition was further translated into an environmental indicator, which is the detoxification of the wastewater (Figure 6B). Interestingly, the ME-FBR operation rapidly decreased the ecotoxicity of the effluent (Figure 6B) regardless of the electrode potential tested. This suggested the absence of toxic metabolites after microbial metabolism of CBZ and SMX. In other words, the high detoxification achieved during the ME-FBR operation denotes the depletion of the environmental risk associated with the initial wastewater polluted with CBZ and SMX after the ME-FBR operation. In addition, among all potential tests, those enhancing oxidative conditions [especially + 400 mV (vs. Ag/Ag)] showed detoxification as high as 70%. We can conclude that such high-value redox polarizations severely impact the removal of toxic metabolites from CBZ and SMX; a similar trend was observed for transforming CBZ and SMX (Figure 2). This high detoxification is clearly due to the action of electroactive bacteria in ME-FBR according to the low CBZ and SMX removal obtained in the electrochemical control test.

## Conclusion

Microbial communities’ stimulation using fluid-like electrodes outperformed traditional UASB treatments to remove emergent pollutants like the antibiotics CBZ and SMX. Neither adsorption nor abiotic electrochemical reactions were shown to be responsible for efficient electrobioremediation. Furthermore, we have explored the system’s bioelectrochemical response, revealing an impact of redox potential not only in biodegrading but also in detoxifying the resulting effluent. The current ME-FBR operation under lab scale represents the first stage of a promising solution for cleaning up wastewater from the pharmaceutical industry.

## Data Availability Statement

The original contributions presented in the study are included in the article/Supplementary Material, further inquiries can be directed to the corresponding author/s.

## Author Contributions

YA designed the study, carried out the majority of the experiments, and drafted the manuscript. ML, AS-G, and CM helped with the experimental work. KB and AE-N supervised the study and corrected the manuscript. All authors read and approved the final manuscript.

## Conflict of Interest

The authors declare that the research was conducted in the absence of any commercial or financial relationships that could be construed as a potential conflict of interest.

## Publisher’s Note

All claims expressed in this article are solely those of the authors and do not necessarily represent those of their affiliated organizations, or those of the publisher, the editors and the reviewers. Any product that may be evaluated in this article, or claim that may be made by its manufacturer, is not guaranteed or endorsed by the publisher.

## References

[B1] Abdel-Shafy H. Mansour M. (2013). Issue of pharmaceutical compounds in water and wastewater: sources, impact and elimination. *Egypt. J. Chem.* 566 449–471.

[B2] Ali A. (2019). Removal of sulfamethoxazole, sulfapyridine and carbamazepine, from simulated wastewater using conventional and nonconventional adsorbents. *Int. J. Environ. Res.* 13 28–30. 10.1007/s41742-019-00192-x

[B3] Angeles L. G. Mullen R. Huang I. Wilson C. Khunjar W. Sirotkin H. (2019). Assessing pharmaceutical removal and reduction in toxicity provided by advanced wastewater treatment systems. *Environ. Sci. Water Res. Technol.* 6 62–77. 10.1039/C9EW00559E

[B4] Asensio Y. Llorente M. Fernández P. Tejedor-Sanz S. Ortiz J. M. Ciriza J. F. (2021). Upgrading fluidized bed bioelectrochemical reactors for treating brewery wastewater by using a fluid-like electrode. *Chem. Eng. J.* 406:127103. 10.1016/j.cej.2020.127103

[B5] Bajracharya S. Sharma M. Mohanakrishna G. Dominguez Benneton X. Strik D. P. B. T. B. Sarma P. M. (2016). An overview on emerging bioelectrochemical systems (BESs): technology for sustainable electricity, waste remediation, resource recovery, chemical production and beyond. *Renew. Energy* 98 153–170. 10.1016/j.renene.2016.03.002

[B6] Busalmen J. P. Esteve-Nuñez A. Feliu J. M. (2008). Whole cell electrochemistry of electricity-producing microorganisms evidence an adaptation for optimal exocellular electron transport. *Environ. Sci. Technol.* 42 2445–2450. 10.1021/es702569y 18504979

[B7] Cecconet D. Molognoni D. Callegari A. Capodaglio A. G. (2017). Biological combination processes for efficient removal of pharmaceutically active compounds from wastewater: a review and future perspectives. *J. Environ. Chem. Eng.* 5 3590–3603. 10.1016/j.jece.2017.07.020

[B8] Cecconet D. Molognoni D. Callegari A. Capodaglio A. G. (2018). Agro-food industry wastewater treatment with microbial fuel cells: energetic recovery issues. *Int. J. Hydrogen Energy* 43 500–511. 10.1016/j.ijhydene.2017.07.231

[B9] Cheng D. Ngo H. H. Guo W. Liu Y. Chang S. W. Nguyen D. D. (2018). Anaerobic membrane bioreactors for antibiotic wastewater treatment: performance and membrane fouling issues. *Bioresour. Technol.* 267 714–724. 10.1016/j.biortech.2018.07.133 30082132

[B10] Chtourou M. Mallek M. Dalmau M. Mamo J. Santos-Clotas E. Salah A. (2018). Triclosan, carbamazepine and caffeine removal by activated sludge system focusing on membrane bioreactor. *Process. Saf. Environ. Prot.* 118 1–9. 10.1016/j.psep.2018.06.019

[B11] Domínguez-Garay A. Boltes K. Esteve-Núñez A. (2016). Cleaning-up atrazine-polluted soil by using microbial electroremediating cells. *Chemosphere* 161 365–371. 10.1016/j.chemosphere.2016.07.023 27448317

[B12] Gadipelly C. Pérez-González A. Yadav G. Ortiz I. Ibáñez R. Rathod V. (2014). Pharmaceutical industry wastewater: review of the technologies for water treatment and reuse. *Ind. Eng. Chem. Res.* 53 11571–11592. 10.1021/ie501210j

[B13] Gajda I. Greenman J. Ieropoulos I. A. (2018). Recent advancements in real-world microbial fuel cell applications. *Curr. Opin. Electrochem.* 11 78–83. 10.1016/j.coelec.2018.09.006 31417973 PMC6686732

[B14] Gao N. Fan Y. Long F. Qiu Y. Geier W. Liu H. (2020). Novel trickling microbial fuel cells for electricity generation from wastewater. *Chemosphere* 248:126058. 10.1016/j.chemosphere.2020.126058 32045974

[B15] García-Espinoza J. D. Nacheva P. M. (2019). Degradation of pharmaceutical compounds in water by oxygenated electrochemical oxidation: parametric optimization, kinetic studies and toxicity assessment. *Sci. Total Environ.* 691 417–429. 10.1016/j.scitotenv.2019.07.118 31323587

[B16] García-Gómez C. Drogui P. Seyhi B. Gortáres-Moroyoqui P. Buelna G. Estrada-Alvgarado M. I. (2016). Combined membrane bioreactor and electrochemical oxidation using Ti/PbO2 anode for the removal of carbamazepine. *J. Taiwan Inst. Chem. Eng.* 64 211–219. 10.1016/j.jtice.2016.04.024

[B17] Gurung K. Ncibi M. C. Shestakova M. Sillanpää M. (2018). Removal of carbamazepine from MBR effluent by electrochemical oxidation (EO) using a Ti/Ta2O5-SnO2 electrode. *Appl. Catal. B Environ.* 221 329–338. 10.1016/j.apcatb.2017.09.017

[B18] Hai F. Li X. Price W. Nghiem L. (2011). Removal of carbamazepine and sulfamethoxazole by MBR under anoxic and aerobic conditions. *Bioresour. Technol.* 102 10386–10390. 10.1016/j.biortech.2011.09.019 21963248

[B19] Harnisch F. Gimkiewicz C. Bogunovic B. Kreuzig R. Schröder U. (2013). On the removal of sulfonamides using microbial bioelectrochemical systems. *Electrochem. Commun.* 26 77–80. 10.1016/j.elecom.2012.10.015

[B20] Hoareau M. Erable B. Bergel A. (2019). Microbial electrochemical snorkels (MESs): a budding technology for multiple applications. A mini review. *Electrochem. Commun.* 104:106473. 10.1016/j.elecom.2019.05.022

[B21] Leon-Fernandez L. F. Villaseñor J. Rodriguez L. Cañizares P. Rodrigo M. A. Fernández-Morales F. J. (2019). Dehalogenation of 2,4-dichlorophenoxyacetic acid by means of bioelectrochemical systems. *J. Electroanal. Chem.* 854:113564. 10.1016/j.jelechem.2019.113564

[B22] Li J. Dodgen L. Ye Q. Gan J. (2013). Degradation kinetics and metabolites of carbamazepine in soil. *Environ. Sci. Technol.* 47 3678–3684. 10.1021/es304944c 23506704

[B23] Lin C.-W. Chen J. Zhao J. Liu S.-H. Lin L.-C. (2020). Enhancement of power generation with concomitant removal of toluene from artificial groundwater using a mini microbial fuel cell with a packed-composite anode. *J. Hazard. Mater.* 387:121717. 10.1016/j.jhazmat.2019.121717 31767505

[B24] Liu H. Grot S. Logan B. E. (2005). Electrochemically assisted microbial production of hydrogen from acetate. *Environ. Sci. Technol.* 39 4317–4320. 10.1021/es050244p 15984815

[B25] Logan B. E. (2008). *Microbial Fuel Cells.* Hoboken, NJ: John Wiley & Sons.

[B26] Mestre A. Carvalho A. (2019). Photocatalytic degradation of pharmaceuticals carbamazepine, diclofenac, and sulfamethoxazole by semiconductor and carbon materials: a review. *Molecules* 24:3702. 10.3390/molecules24203702 31618947 PMC6832631

[B27] Nassiri Koopaei N. Abdollahi M. (2017). Health risks associated with the pharmaceuticals in wastewater. *Daru* 25:9. 10.1186/s40199-017-0176-y 28403898 PMC5389172

[B28] Pun Á Boltes K. Letón P. Esteve-Nuñez A. (2019). Detoxification of wastewater containing pharmaceuticals using horizontal flow bioelectrochemical filter. *Bioresour. Technol. Rep.* 7:100296. 10.1016/j.biteb.2019.100296

[B29] Queiroz M. T. A. Queiroz C. A. Alvim L. B. Sabará M. G. Leão poundso M. M. D. de Amorim C. C. (2019). *ReestruturaÃ§Ã\poundso na Forma do Tratamento de Efluentes tÃ\Textordfemeninexteis: Uma Proposta Embasada em Fundamentos Teã\Textthreesuperiorricos. GestÃ\poundso & ProduÃ§Ã\poundso 26.* Available online at: http://www.scielo.br/scielo.php?script=sci_arttext&pid=S0104-530X2019000100215&nrm=iso.

[B30] Ramírez-Vargas C. A. Arias C. A. Carvalho P. Zhang L. Esteve-Núñez A. Brix H. (2019). Electroactive biofilm-based constructed wetland (EABB-CW): a mesocosm-scale test of an innovative setup for wastewater treatment. *Sci. Total Environ.* 659 796–806. 10.1016/j.scitotenv.2018.12.432 31096410

[B31] Rodrigo M. A. Cañizares P. Sánchez-Carretero A. Sáez C. (2010). Use of conductive-diamond electrochemical oxidation for wastewater treatment. *Catal. Today* 151 173–177. 10.1016/j.cattod.2010.01.058

[B32] Rodrigo Quejigo J. Tejedor-Sanz S. Schroll R. Esteve-Núñez A. (2019). Electrodes boost microbial metabolism to mineralize antibiotics in manure. *Bioelectrochemistry* 128 283–290. 10.1016/j.bioelechem.2019.04.008 31059968

[B33] Sara T.-S. Patricia F.-L. Carlos M. Abraham E.-N. (2020). Fluidized bed cathodes as suitable electron donors for bacteria to remove nitrogen and produce biohydrogen. *Electrochem. Commun.* 116:106759. 10.1016/j.elecom.2020.106759

[B34] Scott K. Yu E. H. (2015). *Microbial Electrochemical and Fuel Cells: Fundamentals and Applications.* Sawston: Woodhead Publishing.

[B35] Song H.-L. Lu Y.-X. Yang X.-L. Xu H. Singh R. P. Du K.-X. (2020). Degradation of sulfamethoxazole in low-C/N ratio wastewater by a novel membrane bioelectrochemical reactor. *Bioresour. Technol.* 305:123029. 10.1016/j.biortech.2020.123029 32109730

[B36] Srikanth S. Marsili E. Flickinger M. C. Bond D. R. (2008). Electrochemical characterization of Geobacter sulfurreducens cells immobilized on graphite paper electrodes. *Biotechnol. Bioeng.* 99 1065–1073. 10.1002/bit.21671 17929324

[B37] Tahir K. Miran W. Nawaz M. Jang J. Shahzad A. Moztahida M. (2019). Investigating the role of anodic potential in the biodegradation of carbamazepine in bioelectrochemical systems. *Sci. Total Environ.* 688 56–64. 10.1016/j.scitotenv.2019.06.219 31229828

[B38] Tejedor-Sanz S. Bacchetti De Gregoris T. Salas J. J. Pastor L. Esteve-Núñez A. (2016). Integrating a microbial electrochemical system into a classical wastewater treatment configuration for removing nitrogen from low COD effluents. *Environ. Sci. Water Res. Technol.* 2 884–893. 10.1039/c6ew00100a

[B39] Tejedor-Sanz S. Fernández-Labrador P. Hart S. Torres C. I. Esteve-Núñez A. (2018). Geobacter dominates the inner layers of a stratified biofilm on a fluidized anode during brewery wastewater treatment. *Front. Microbiol.* 9:378. 10.3389/fmicb.2018.00378 29568284 PMC5853052

[B40] Tejedor-Sanz S. Ortiz J. M. Esteve-Núñez A. (2017a). Merging microbial electrochemical systems with electrocoagulation pretreatment for achieving a complete treatment of brewery wastewater. *Chem. Eng. J.* 330 1068–1074. 10.1016/j.cej.2017.08.049

[B41] Tejedor-Sanz S. Quejigo J. R. Berná A. Esteve-Núñez A. (2017b). The planktonic relationship between fluid-like electrodes and bacteria: wiring in motion. *ChemSusChem* 10 693–700. 10.1002/cssc.201601329 27860438

[B42] Tiquia-Arashiro S. Pant D. (2020). *Microbial Electrochemical Technologies.* Milton Park: Routledge.

[B43] Viggi C. C. Matturro B. Frascadore E. Insogna S. Mezzi A. Kaciulis S. (2017). Bridging spatially segregated redox zones with a microbial electrochemical snorkel triggers biogeochemical cycles in oil-contaminated River Tyne (UK) sediments. *Water Res.* 127 11–21. 10.1016/j.watres.2017.10.002 29020640

[B44] Virkutyte J. Varma R. Jegatheesan V. (2010). *Treatment of Micropollutants in Water and Wastewater.* New York, NY: IWA Publishing.

[B45] Wang X. Aulenta F. Puig S. Esteve-Núñez A. He Y. Mu Y. (2020). Microbial electrochemistry for bioremediation. *Environ. Sci. Ecotechnol.* 1:100013. 10.1016/j.ese.2020.100013 PMC9488016 36160374

[B46] Yakubu O. H. (2017). Pharmaceutical wastewater effluent-source of contaminants of emerging concern: phytotoxicity of metronidazole to soybean (glycine max). *Toxics* 5:10. 10.3390/toxics5020010 29051442 PMC5606667

[B47] Yang Q. Zhao H. Liang H. (2015). Denitrification of overlying water by microbial electrochemical snorkel. *Bioresour. Technol.* 197 512–514. 10.1016/j.biortech.2015.08.127 26362461

[B48] Yang Y. Guo J. Sun G. Xu M. (2013). Characterizing the snorkeling respiration and growth of Shewanella decolorationis S12. *Bioresour. Technol.* 128 472–478. 10.1016/j.biortech.2012.10.103 23201531

[B49] Zhang Y. Angelidaki I. (2014). Microbial electrolysis cells turning to be versatile technology: recent advances and future challenges. *Water Res.* 56 11–25. 10.1016/j.watres.2014.02.031 24631941

[B50] Zhang Y. Geissen S.-U. Gal C. (2008). Carbamazepine and diclofenac: removal in wastewater treatment plants and occurrence in water bodies. *Chemosphere* 73 1151–1161. 10.1016/j.chemosphere.2008.07.086 18793791

