# Supplementary figures and images for: Microbial Electrochemical Fluidized Bed Reactor: A Promising Solution for Removing Pollutants From Pharmaceutical Industrial Wastewater
Source: Front Microbiol. 2021 Nov 26;12:737112. doi: 10.3389/fmicb.2021.737112 (PMC8664407; doi:10.3389/fmicb.2021.737112)

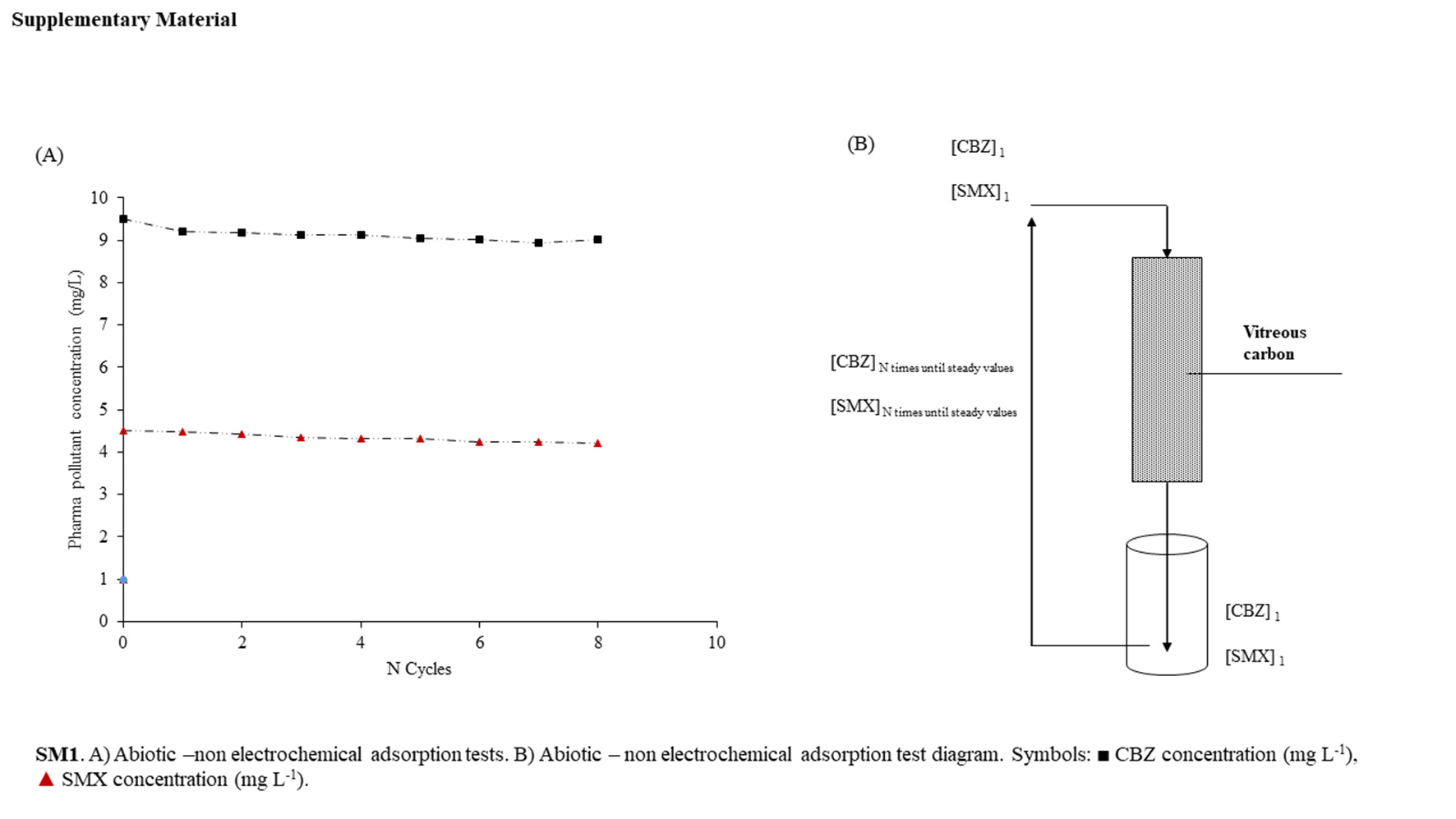

Supplement: Supplementary file 1 [file Image_1.TIF]

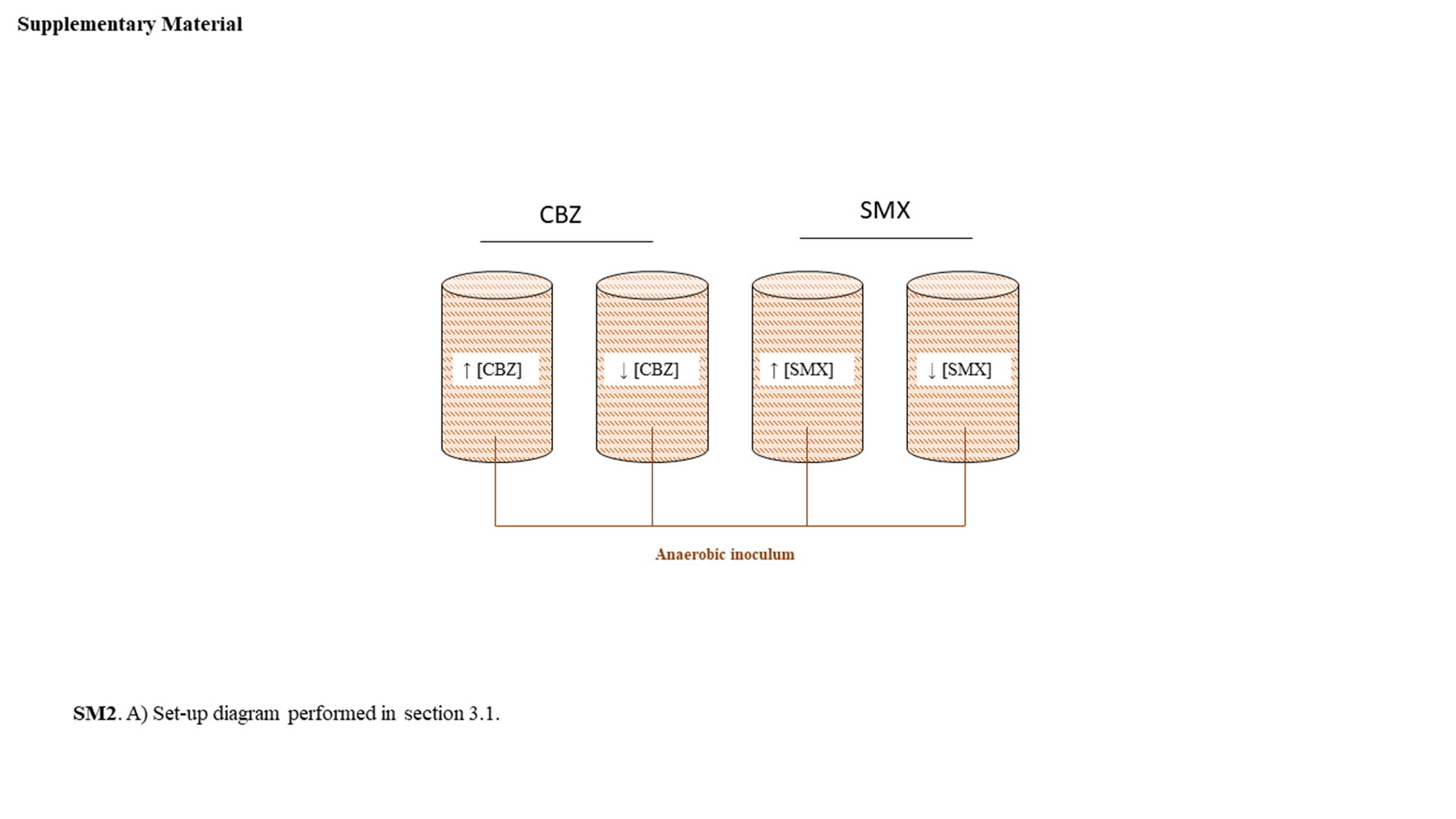

Supplement: Supplementary file 2 [file Image_2.TIF]

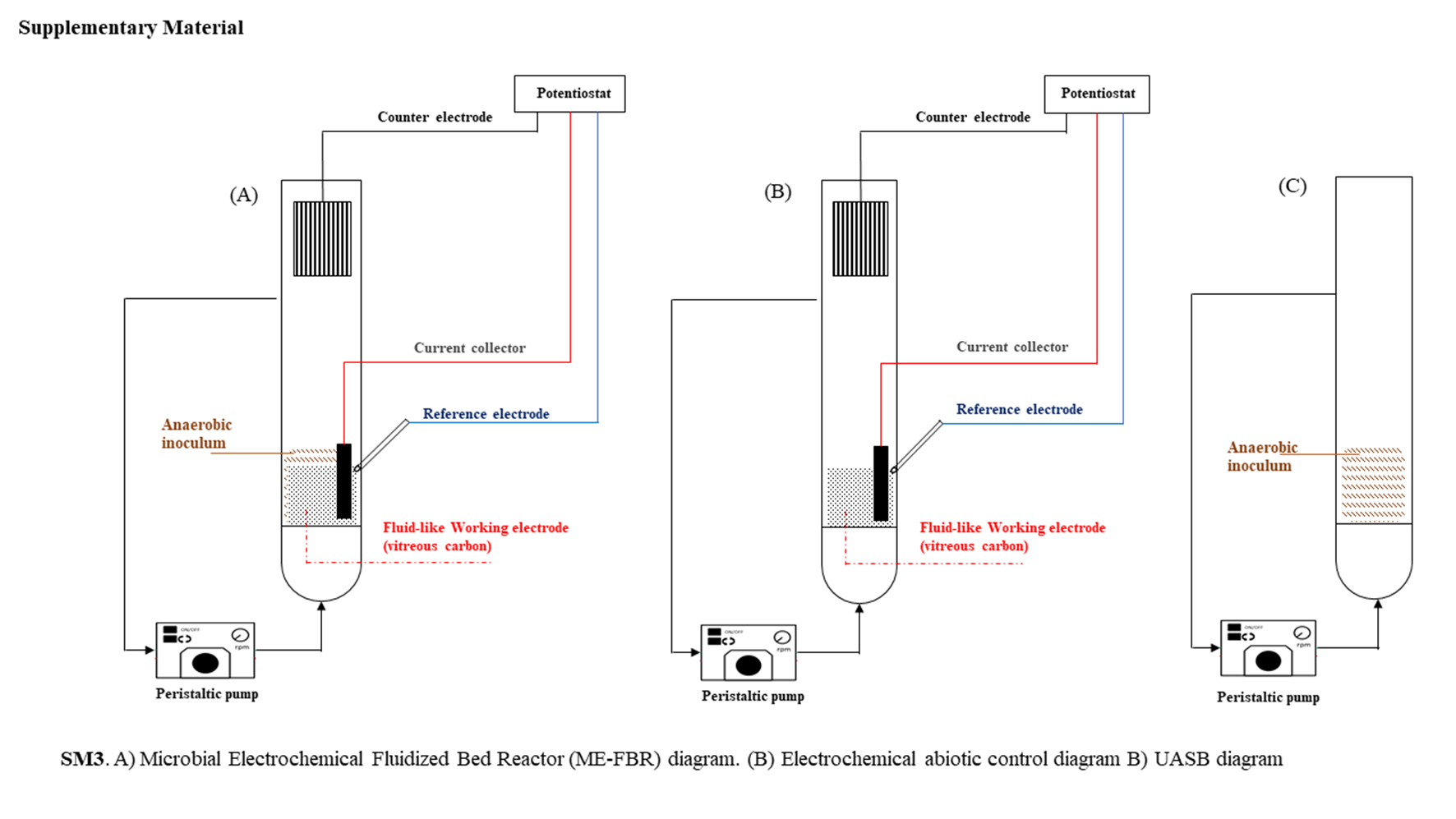

Supplement: Supplementary file 3 [file Image_3.TIF]
